# Antimicrobial Resistance Profile in ICU Patients Across India: A Multicenter, Retrospective, Observational Study

**DOI:** 10.7759/cureus.57489

**Published:** 2024-04-02

**Authors:** Vandana Verma, Chithra Valsan, Preety Mishra, Kalpana Mund, Soma Dutta, Geethanjali Anke, Harsha Sasi, Dhara Shah

**Affiliations:** 1 Department of Microbiology, Christian Medical College & Hospital, Ludhiana, IND; 2 Department of Microbiology, Jubilee Mission Medical College and Research Institute, Thrissur, IND; 3 Department of Microbiology, Kalinga Institute of Medical Sciences, Bhubaneswar, IND; 4 Department of Microbiology, Apollo Multispeciality Hospitals, Kolkata, IND; 5 Department of Microbiology, KIMS SAVEERA Hospital, Anantapur, IND; 6 Department of Medical Affairs, Viatris, India, Bangalore, IND

**Keywords:** mrsa, eskape, gram negative bacteria, gram positive bacteria, antimicrobial resistance

## Abstract

Background

The pattern of antimicrobial resistance (AMR) changes with time and varies in countries and between hospitals within the same country. Physicians might thus benefit from information on regional resistance patterns of clinically significant bacterial isolates when deciding on the best empirical treatment. Numerous nosocomial infections are caused by multidrug-resistant (MDR) strains, notably methicillin-resistant Staphylococcus aureus (MRSA) strains, which are also linked to higher morbidity and death.

Aim

Evaluation of AMR profile in intensive care unit (ICU) patients of multiple tertiary care centers across India.

Methods

This was a multicenter, retrospective study based on electronic laboratory records of microbial isolates from clinical specimens from ICUs analyzed at microbiology laboratories of identified hospitals. Data of invasive sample records was collected from Microbiology labs of the identified hospitals within India and were aligned to WHO 5 Net standard reporting and as per Clinical & Laboratory Standards Institute (CLSI-2014) Guidelines for assessment. Data from 21556 samples were collected retrospectively from December 2021 to January 2010. Antibiotic susceptibility testing was done by using both the Kirby Baur disk diffusion method and the automated method (using the Vitek 2 compact system) as per CLSI (2014) guidelines.

Results

Of 21,556 enrolled patients, the majority (54.12%) were males and adults (62.07%). The median age was 58 years. Of 815 gram-positive bacteria reports, the commonest were S. aureus (552, 67.73%), Coagulase-negative Staphylococci (107, 13.13%), and Enterococcus spp. (105, 12.88%). For Coagulase-negative Staphylococci-positive samples, resistance was to penicillin (79, 73.83%), and erythromycin (73, 68.22%); and for S. aureus was to ciprofloxacin (361, 65.4%), and erythromycin (315,57.07%). Enterococcus spp. showed maximum resistance to erythromycin (73, 69.52%), followed by ampicillin, ciprofloxacin (68,64.76% each). Of 4,183 gram-negative bacteria reports, the commonest were Klebsiella pneumoniae (1,531, 36.6%), Escherichia coli (1,269, 30.34%), and Acinetobacter spp. (589, 14.08%), Pseudomonas aeruginosa (438, 14.08%), other Klebsiella spp. (174, 4.16%) and Enterobacter spp. (161, 3.85%). K. pneumoniae showed resistance to ciprofloxacin (1,001, 65.38%). E. coli showed resistance to ampicillin (918, 72.34%), and ciprofloxacin (798,62.88%); and Acinetobacter spp. to ceftazidime (525, 89.13%), and ciprofloxacin (507, 86.08%), while P. aeruginosa showed resistance to imipenem (234, 53.42%). Enterobacter spp. showed resistance to cefotaxime (129, 80.12%). MRSA samples showed resistance to phenoxymethylpenicillin (188, 35.54%) and benzylpenicillin (178, 33.46%).

Conclusion

Gram-negative bacteria were more common than gram-positive bacteria in causing antibiotic-resistant infections in ICU, with beta-lactams, fluoroquinolones, macrolides, and cephalosporins showing varied percentages of resistance. Fluoroquinolones, macrolides, and penicillin were noted to be highly resistant against gram-positive species. This indicates that evaluation based on MDR and antibiotic consumption patterns is imperative.

## Introduction

Overuse of antibiotic agents is a public health problem associated with increased healthcare costs and antimicrobial resistance (AMR), which is considered a major threat to global health, and has burdened low and middle-income countries [1,2]. Contributing factors involve patients' exposure to invasive bacterial illness, unrestrained usage of antibiotics, and poor laboratory patronage for diagnosis leading to antibiotic overuse. The choice, dose, route, and duration of antimicrobial therapy must be tailored to each patient-by-patient characteristics, severity of illness, potential infecting organisms, and local resistance patterns [3]. Most physicians know with regards to contributory factors to antibiotic resistance, but appropriate antibiotic selection is not reflected many times [4].

Antibiotic resistance can be reduced by implementing evidence-based practices [5]. The pattern of AMR changes with time and varies across countries and between hospitals within the same country. Physicians might thus benefit from information on regional resistance patterns of clinically significant bacterial isolates when deciding on the best empirical treatment.

Staphylococcus aureus is a major pathogen in both the hospital environment and the wider community. It is a Gram-positive bacterium living on the skin, mouth, and upper respiratory system, making it a risk factor for opportunistic and nosocomial infections [6]. Invasive infections are frequently associated with life-threatening bacteremia infections [7]. Numerous nosocomial infections are caused by multidrug-resistant (MDR) strains, notably methicillin-resistant S. aureus (MRSA) strains, which are also linked to higher morbidity and death [8]. Horizontal gene transfer in the hospital setting is responsible for disseminating antibiotic-resistant determinants.

Enterococcus faecium, S. aureus, Klebsiella pneumoniae, Acinetobacter baumannii, Pseudomonas aeruginosa, and Enterobacter species (ESKAPE) include both Gram-positive and Gram-negative bacteria. These bacteria frequently cause serious nosocomial infections in immunocompromised and critically sick patients [9].

AMR Surveillance has been identified by the World Health Organization (WHO) for containment of AMR which plays a significant role in the Indian subcontinent with India being one of the largest consumers of antibiotics in the world [10, 11]. Additionally, factors including a greater illness load, inadequate public health infrastructure, growing wealth, and unrestricted drug sales have exacerbated AMR [12]. Systematic nationwide surveillance of AMR pathogens in India is inadequate and the published AMR surveillance report by the WHO from 169 member countries mentioned a lack of national surveillance data on resistant pathogens in India and 14 other member countries [13]. The aim of the present study was to evaluate the AMR profile at ICUs of multiple tertiary care centers across India.

## Materials and methods

Study design

**Figure 1:**
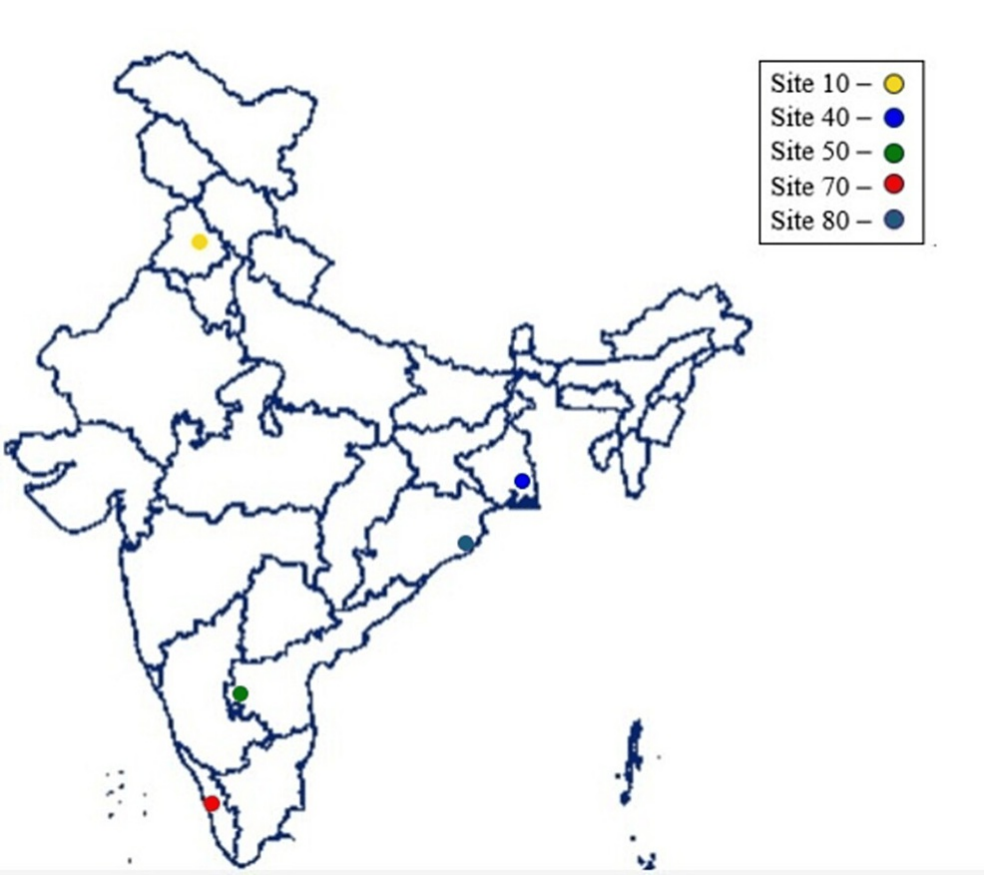
Study sites across India. The base map of India can be found on https://bharatmaps.gov.in/BharatMaps/Home/Map

This was a multicentric, retrospective study based on laboratory records of microbial isolates from clinical specimens from ICUs analyzed at microbiology laboratories during 2010-2021 of the identified hospitals to evaluate the antimicrobials resistance profile in ICU patients. From South India, Jubilee Hospital (1,750 beds), Kerala (Site 70); KIMS SAVEERA (250 beds), Andhra Pradesh (Site 50); from East India, Kalinga Institute of Medical Sciences (2,600 bed), Odisha (Site 80); Apollo Gleneagles (700 bed), West Bengal (Site 40), and from North India, Dayanand Medical College Hospital (1,326 beds), Punjab (Site 10) were included (Figure 1). The isolates that were reported as colonizers were excluded, and so were those from swabs or aspirates sent as samples. The study protocol was approved by the Institutional Ethics Committee of Apollo Gleneagles Hospital Limited, Jubilee Mission Medical College and Research Institute, Kalinga Institute of Medical Sciences, and the S2J Independent Ethics Committee. Informed consent waiver was obtained from the Ethics Committee as this was a retrospective study.

Data collection and Interpretation

Data of invasive sample records was collected from Microbiology labs of the identified hospitals and were aligned to WHO 5 Net standard reporting and as per Clinical & Laboratory Standards Institute (CLSI) Guidelines for assessment. Data of 21556 samples were collected. Past records were obtained, and antibiotic susceptibility testing was done by using both Kirby Baur disk diffusion method and automated method (using Vitek 2 compact system) as per CLSI (2014) guidelines. The antibiotics were selected according to the clinical samples as recommended by CLSI. Antimicrobial susceptibility results were collected, entered in database, verified, and analyzed. The rates of susceptibility to individual antimicrobials were calculated for every bacterial isolate.

Statistical analysis

Descriptive statistics were used. Combined antimicrobial drug susceptibility data were analyzed for the target organisms. Prevalence of AMR across India was calculated. The mean percentages of the susceptibility and resistance of each isolate to all tested antimicrobials were calculated. The overall rates of susceptibility and resistance pattern for common isolated gram-positive and gram-negative organisms were calculated. Microbiologists provided the raw data, errors and quality check was carried out by clinical data associate and the statistician performed the analysis. The analysis was done using Statistical Package for the Social Sciences (SPSS) Statistics version 20.0 (IBM Corp., Armonk, NY).

## Results

The total number of subjects enrolled in the study was 21,556. Of these, the culture of 8,377 (38.86%) specimens showed growth of the organism, while the culture of 13,179 (61.14%) specimens did not show any growth of bacteria.

Demographic characteristics

Of the total enrolled patients (N=21,556), 11,667 (54.12%) were males and 4,408 (20.45%) were females. Gender data of 5,481 (25.43%) cases were missing. 2,635 (12.22%) of the enrolled cases were newborn, 502 (2.33%) were infants, 936 (4.34%) were belonging to the age-group 1-15 years, and 13,379 (62.07%) were adults. Age details were noted for 10,782 of the enrolled cases. The mean±standard deviation (SD) age was found to be 55.2±20.38 years. The median age was 58 years, with inter-quartile range (IQR) of 44-69 years.

AMR profile of frequently isolated Gram-positive bacteria

From the 815 culture reports of commonly isolated gram-positive bacteria, 552 (67.73%) samples were S. aureus, 107 (13.13%) samples were Coagulase-negative Staphylococci, 105 (12.88%) were Enterococcus spp., Other isolated organisms were Streptococcus agalactiae 22 (2.7%), S. pneumoniae 21 (2.58%), Streptococcus pyogenes five (0.61%), and Streptococcus viridans three (0.37%).

For Coagulase-negative Staphylococci, the commonest resistance was penicillin 79 (73.83%), followed by erythromycin 73 (68.22%), ciprofloxacin 52 (48.6%), ampicillin 45 (42.06%). The commonest resistance noted by S. aureus was for ciprofloxacin (n=361, 65.4%), followed by erythromycin 315 (57.07%) and trimethoprim-sulfamethoxazole 199 (36.05%). Overall, the proportion of S. aureus cases with AMR was 96.37% and 3.8% were resistant to vancomycin. For MRSA samples, the commonest noted resistance was to phenoxymethylpenicillin 188 (35.54%), followed by benzylpenicillin 178(33.46%). Cefoxitin showed resistance to 13.22% samples of S. aureus and 13.72% of MRSA, respectively. Enterococcus spp. showed maximum resistance to erythromycin 73 (69.52%), followed by ampicillin, ciprofloxacin 68(64.76%) each, gentamicin 59(56.19%) and phenoxymethylpenicillin 58 (55.24%). 21.9% of enterococci were also resistant to vancomycin (VRE). For Streptococcus agalactiae, the commonest resistance was noted for tetracycline 10(45.45%). S. pneumoniae positive samples showed commonest resistance for trimethoprim-sulfamethoxazole 15 (71.43%). For Streptococcus pyogenes, two of five samples (40%) were resistant to erythromycin, while for Streptococcus viridans, two samples were resistant to ampicillin (Table 1).

**Table 1:** Antimicrobial resistance profile of frequently isolated gram-positive bacteria, India Abbreviations: N = Number of subjects in specified pathogen; n = Number of resistant bacterial isolates. Percentages of resistant bacterial isolates are based on the number of subjects in specified pathogen. * Common resistance profiles.

Antibacterial agent	Coagulase-negative Staphylococcus N = 107 n (%)	Staphylococcus aureus N = 552 n (%)	Enterococcus spp. N = 105 n (%)	Streptococcus agalactiae N = 22 n (%)	Streptococcus pyogenes N = 5 n (%)	Streptococcus pneumoniae N = 21 n (%)	Streptococcus viridans group N = 3 n (%)
Amikacin	11 (10.28)	10 (1.81)	4 (3.81)	0	0	1 (4.76)	0
Amoxicillin	16 (14.95)	16 (2.90)	7 (6.67)	0	0	3 (14.29)	1 (33.33)^ *^
Amoxicillin-Clavulanate	29 (27.10)^*^	87 (15.76)	9 (8.57)	0	0	0	0
Ampicillin	45 (42.06)^ *^	82 (14.86)	68 (64.76)^*^	0	0	3 (14.29)	2 (66.67)^ *^
Ampicillin­Sulbactam	13 (12.15)	14 (2.54)	5 (4.76)	0	0	1 (4.76)	0
Azithromycin	0	0	3 (2.86)	0	0	5 (23.81)^ *^	0
Benzylpenicillin	0	178 (32.25)^ *^	1 (0.95)	0	0	0	0
Cefaclor	0	1 (0.18)	0	0	0	0	0
Cefepime	12 (11.21)	10 (1.81)	10 (9.52)	0	0	0	0
Cefixime	13 (12.15)	12 (2.17)	6 (5.71)	0	0	1 (4.76)	0
Cefoperazone-Sulbactam	13 (12.15)	11 (1.99)	9 (8.57)	0	0	1 (4.76)	0
Cefotaxime	11 (10.28)	10 (1.81)	14 (13.33)	0	0	2 (9.52)	1 (33.33)
Cefoxitin	24 (22.43)^ *^	73 (13.22)	2 (1.90)	0	0	2 (9.52)	0
Ceftazidime	13 (12.15)	8 (1.45)	14 (13.33)	0	0	2 (9.52)	0
Ceftriaxone	14 (13.08)	12 (2.17)	14 (13.33)	0	0	2 (9.52)	1 (33.33)
Chloramphenicol	1 (0.93)	0	3 (2.86)	0	0	0	0
Ciprofloxacin	52 (48.60)^ *^	361 (65.40)^ *^	68 (64.76)^ *^	1 (4.55)	0	2 (9.52)	0
Clarithromycin	0	18 (3.26)	0	0	0	0	0
Clindamycin	40 (37.38)^ *^	189 (34.24)^ *^	4 (3.81)	1 (4.55)	0	6 (28.57)^ *^	0
Colistin	3 (2.80)	16 (2.90)	2 (1.90)	0	0	0	0
Doxycycline	13 (12.15)	8 (1.45)	5 (4.76)	0	0	2 (9.52)	0
Ertapenem	10 (9.35)	12 (2.17)	1 (0.95)	0	0	1 (4.76)	0
Erythromycin	73 (68.22)^ *^	315 (57.07)^ *^	73 (69.52)^ *^	1 (4.55)	2 (40.00)^ *^	5 (23.81)^ *^	1 (33.33)
Gatifloxacin	1 (0.93)	0	1 (0.95)	0	0	0	0
Gentamicin	36 (33.64)^ *^	63 (11.41)	59 (56.19)^ *^	0	0	1 (4.76)	1 (33.33)
Imipenem	9 (8.41)	7 (1.27)	8 (7.62)	0	0	0	0
Levofloxacin	3 (2.80)	173 (31.34)^ *^	4 (3.81)	5 (22.73)^ *^	0	2 (9.52)	0
Linezolid	2 (1.87)	27 (4.89)	4 (3.81)	0	0	1 (4.76)	0
Meropenem	12 (11.21)	13 (2.36)	10 (9.52)	0	0	1 (4.76)	0
Minocycline	1 (0.93)	0	0	0	0	0	0
Netilmicin	1 (0.93)	0	2 (1.90)	0	0	0	0
Nitrofurantoin	3 (2.80)	16 (2.90)	8 (7.62)	0	0	0	0
Norfloxacin	7 (6.54)	5 (0.91)	7 (6.67)	0	0	0	0
Ofloxacin	5 (4.67)	3 (0.54)	11 (10.48)	0	0	2 (9.52)	0
Oxacillin	6 (5.61)	80 (14.49)	0	0	0	0	0
Phenoxymethylpenicillin	79 (73.83)^ *^	188 (34.06)^ *^	58 (55.24)^ *^	0	0	1 (4.76)	1 (33.33)
Piperacillin	3 (2.80)	82 (14.86)	5 (4.76)	1 (4.55)	0	0	0
Piperacillin-Tazobactam	10 (9.35)	11 (1.99)	7 (6.67)	0	0	0	0
Rifampicin	9 (8.41)	37 (6.70)	0	0	0	0	0
Teicoplanin	1 (0.93)	16 (2.90)	24 (22.86)^ *^	3 (13.64)	0	1 (4.76)	0
Tetracycline	11 (10.28)	33 (5.98)	7 (6.67)	10 (45.45)^ *^	1 (20.00)^ *^	1 (4.76)	0
Ticarcillin	0	0	2 (1.90)	0	0	0	0
Tigecycline	4 (3.74)	4 (0.72)	1 (0.95)	0	0	0	0
Tobramycin	0	0	1 (0.95)	0	0	0	0
Trimethoprim	17 (15.89)	10 (1.81)	0	1 (4.55)	0	0	0
Trimethoprim-Sulfamethoxazole	30 (28.04)^ *^	199 (36.05)^ *^	7 (6.67)	3 (13.64)	1 (20.00)^ *^	15 (71.43)^ *^	0
Vancomycin	1 (0.93)	21 (3.80)	23 (21.90)^ *^	0	0	0	0
Other	0	0	2 (1.90)	0	0	0	0
Range (%)	1-74	0-65	1-70	5-45	20-40	5-71	33-67

AMR profile of frequently isolated Gram-negative bacteria

From the culture reports of 4,183 samples, the commonest gram-negative bacteria isolated was K. pneumoniae 1,531 (36.6%), followed by E. coli 1,269 (30.34%), Acinetobacter spp. 589 (14.08%), P. aeruginosa 438 (14.08%), other Klebsiella spp. 174 (4.16%), Enterobacter spp. 161 (3.85%), Morganella morganii 12 (0.29%), Citrobacter spp. six (0.14%), and Proteus spp. three (0.07%).

For K. pneumoniae, commonest resistance was to ciprofloxacin 1,001 (65.38%), cefuroxime 931 (60.81%), and cefepime 925 (60.42%). The commonest resistance of E. coli was ampicillin 918 (72.34%), followed by ciprofloxacin 798 (62.88%), ceftriaxone 791 (32.33%). Acinetobacter spp. showed commonest resistance to ceftazidime 525(89.13%), followed by ciprofloxacin 507 (86.08%), gentamicin 501 (85.06%), trimethoprim-sulfamethoxazole 454 (77.08%), amikacin 425 (72.16%), and piperacillin-tazobactam 417 (70.80%). For P. aeruginosa, the commonest resistance noted was to imipenem 234 (53.42%) and meropenem 227 (51.83%). Enterobacter spp. showed resistance most commonly to cefotaxime 129 (80.12%), ofloxacin 122 (75.78%), cefoperazone 120 (74.53%), ceftriaxone 119 (73.91%), and ceftazidime 117 (72.67%). Ten out of 12 (83.33%) Morganella morganii showed resistance to ampicillin and cefuroxime. All six samples positive for Citrobacter spp. were resistant to cefotaxime, cefixime, amoxicillin, amoxicillin-clavulanate, and piperacillin-tazobactam. Two out of three (66.67%) Proteus spp. were resistant to amikacin, ampicillin, cefoperazone, cefoperazone-sulbactam, cefotaxime, ceftazidime, ceftriaxone, imipenem, meropenem, ofloxacin, piperacillin-tazobactam, and trimethoprim-sulfamethoxazole (Table 2).

**Table 2:** Antimicrobial resistance profile of frequently isolated gram-negative bacteria, India Abbreviations: N = Number of subjects in specified pathogen; n = Number of resistant bacterial isolates. Percentages of resistant bacterial isolates are based on the number of subjects in specified pathogen. * Common resistance profiles.

Antibacterial agent	Klebsiella Pneumoniae N = 1531 n (%)	Klebsiella spp N = 174 n (%)	Escherichia Coli N = 1269 n (%)	Enterobacter spp N = 161 n (%)	Proteus spp N = 3 n (%)	Citrobacter spp N = 6 n (%)	Pseudomonas aeruginosa N = 438 n (%)	Acinetobacter spp N = 589 n (%)	Morganella morganii N = 12 n (%)
Amikacin	620 (40.50)^ *^	81 (46.55)^ *^	295 (23.25)^ *^	83 (51.55)^ *^	2 (66.67)^ *^	2 (33.33)^ *^	166 (37.90)^ *^	425 (72.16)^ *^	3 (25.00)^ *^
Amoxicillin	191 (12.48)	0	248 (19.54)	3 (1.86)	0	6 (100)	45 (10.27)	13 (2.21)	4 (33.33)^ *^
Amoxicillin-Clavulanate	599 (39.12)^ *^	0	406 (31.99)^ *^	5 (3.11)	0	6 (100)	21 (4.79)	16 (2.72)	7 (58.33)^ *^
Ampicillin	-	110 (63.22)^ *^	918 (72.34)^ *^	-	2 (66.67)^ *^	5 (83.33)^ *^	87 (19.86)	354 (60.10)^ *^	10 (83.33)^ *^
Ampicillin­sulbactam	316 (20.64)	0	160 (12.61)	2 (1.24)	0	3 (50.00)^ *^	21 (4.79)	21 (3.57)	0
Azithromycin	0	5 (2.87)	5 (0.39)	3 (1.86)	0	0	1 (0.23)	137 (23.26)^ *^	0
Aztreonam	265 (17.31)	1 (0.57)	55 (4.33)	1 (0.62)	0	0	86 (19.63)	157 (26.66)^ *^	0
Benzylpenicillin	0	0	0	1 (0.62)	0	0	2 (0.46)	0	0
Cefaclor	0	1 (0.57)	3 (0.24)	0	0	0	0	0	0
Cefadroxil	0	0	1 (0.08)	0	0	0	0	0	0
Cefazolin	15 (0.98)	0	2 (0.16)	0	0	0	0	1 (0.17)	0
Cefepime	925 (60.42)^ *^	59 (33.91)^ *^	652 (51.38)^ *^	36 (22.36)	1 (33.33)^ *^	4 (66.67)^ *^	183 (41.78)^ *^	395 (67.06)^ *^	3 (25.00)
Cefepime-Tazobactam	0	0	0	0	0	0	0	5 (0.85)	0
Cefixime	220 (14.37)	2 (1.15)	264 (20.80)	7 (4.35)	0	6 (100)	16 (3.65)	16 (2.72)	2 (16.67)
Cefoperazone	81 (5.29)	76 (43.68)^ *^	191 (15.05)	120 (74.53)^ *^	2 (66.67)^ *^	0	35 (7.99)	258 (43.80)^ *^	1 (8.33)
Cefoperazone-Sulbactam	779 (50.88)^ *^	87 (50.00)^ *^	440 (34.67)^ *^	92 (57.14)^ *^	2 (66.67)^ *^	3 (50.00)^ *^	159 (36.30)^ *^	309 (52.46)^ *^	4 (33.33)^ *^
Cefotaxime	191 (12.48)	129 (74.14)^ *^	508 (40.03)^ *^	129 (80.12)^ *^	2 (66.67)^ *^	6 (100)^ *^	44 (10.05)	400 (67.91)^ *^	2 (16.67)
Cefoxitin	50 (3.27)	28 (16.09)	76 (5.99)	47 (29.19)	1 (33.33)	0	21 (4.79)	113 (19.19)	0
Cefpodoxime	1 (0.07)	0	0	0	0	0	0	1 (0.17)	0
Ceftaroline	1 (0.07)	0	1 (0.08)	0	0	0	0	0	0
Ceftazidime	319 (20.84)	94 (54.02)^ *^	541 (42.63)^ *^	117 (72.67)^ *^	2 (66.67)^ *^	4 (66.67)^ *^	208 (47.49)^ *^	525 (89.13)^ *^	2 (16.67)
Ceftriaxone	867 (56.63)^ *^	87 (50.00)^ *^	791 (62.33)^ *^	119 (73.91)^ *^	2 (66.67)^ *^	4 (66.67)^ *^	70 (15.98)	387 (65.70)^ *^	2 (16.67)
Ceftriaxone/ Sulbactam/ Edta	0	2 (1.15)	0	4 (2.48)	1 (33.33)	0	1 (0.23)	0	0
Cefuroxime	931 (60.81)^ *^	39 (22.41)	641 (50.51)^ *^	8 (4.97)	0	3 (50.00)^ *^	43 (9.82)	9 (1.53)	10 (83.33)^ *^
Cefuroxime Axetil	394 (25.73)	0	210 (16.55)	0	0	0	0	0	4 (33.33)
Chloramphenicol	298 (19.46)	52 (29.89)^ *^	74 (5.83)	54 (33.54)^ *^	0	0	36 (8.22)	298 (50.59)^ *^	0
Ciprofloxacin	1001 (65.38)^ *^	114 (65.52)^ *^	798 (62.88)^ *^	116 (72.05)^ *^	1 (33.33)	0	189 (43.15)^ *^	507 (86.08)^ *^	7 (58.33)^ *^
Clarithromycin	1 (0.07)	0	0	0	0	0	0	0	0
Clindamycin	3 (0.20)	0	1 (0.08)	0	0	0	2 (0.46)	0	0
Colistin	168 (10.97)	10 (5.75)	56 (4.41)	4 (2.48)	1 (33.33)	0	68 (15.53)	7 (1.19)	-
Dicloxacillin	1 (0.07)	0	0	0	0	0	0	0	0
Doripenem	38 (2.48)	2 (1.15)	27 (2.13)	6 (3.73)	0	0	144 (32.88)^ *^	14 (2.38)	0
Doxycycline	181 (11.82)	0	150 (11.82)	2 (1.24)	0	2 (33.33)^ *^	13 (2.97)	52 (8.83)	-
Ertapenem	892 (58.26)^ *^	4 (2.30)	248 (19.54)	5 (3.11)	0	2 (33.33)	20 (4.57)	20 (3.40)	1 (8.33)
Erythromycin	24 (1.57)	0	3 (0.24)	4 (2.48)	0	0	3 (0.68)	2 (0.34)	0
Fosfomycin	0	0	2 (0.16)	0	0	0	0	1 (0.17)	0
Furazolidone	0	0	10 (0.79)	2 (1.24)	0	0	0	0	0
Gatifloxacin	0	0	2 (0.16)	0	0	0	0	3 (0.51)	0
Gentamicin	908 (59.31)^ *^	95 (54.60)^ *^	500 (39.40)^ *^	105 (65.22)^ *^	1 (33.33)^ *^	2 (33.33)^ *^	178 (40.64)^ *^	501 (85.06)^ *^	1 (8.33)
Imipenem	780 (50.95)^ *^	57 (32.76)^ *^	319 (25.14)^ *^	86 (53.42)^ *^	2 (66.67)^ *^	2 (33.33)^ *^	234 (53.42)^ *^	386 (65.53)^ *^	4 (33.33)^ *^
Kanamycin	0	0	0	0	0	0	0	1 (0.17)	0
Levofloxacin	333 (21.75)^ *^	0	98 (7.72)	5 (3.11)	0	0	157 (35.84)^ *^	177 (30.05)^ *^	0
Linezolid	0	0	0	1 (0.62)	0	0	0	0	0
Mecillinam	3 (0.20)	0	1 (0.08)	0	0	0	0	1 (0.17)	0
Meropenem	1004 (65.58)^ *^	76 (43.68)^ *^	320 (25.22)^ *^	98 (60.87)^ *^	2 (66.67)^ *^	2 (33.33)^ *^	227 (51.83)^ *^	351 (59.59)^ *^	1 (8.33)
Minocycline	24 (1.57)	1 (0.57)	24 (1.89)	0	0	0	20 (4.57)	121 (20.54)^ *^	0
Nalidixic Acid	585 (38.21)^ *^	6 (3.45)	441 (34.75)^ *^	11 (6.83)	0	0	37 (8.45)	5 (0.85)	6 (50.00)^ *^
Netilmicin	104 (6.79)	61 (35.06)^ *^	59 (4.65)	75 (46.58)^ *^	1 (33.33)^ *^	0	22 (5.02)	262 (44.48)^ *^	0
Nitrofurantoin	560 (36.58)^ *^	8 (4.60)	133 (10.48)	28 (17.39)	2 (66.67)	2 (33.33)	-	13 (2.21)	-
Norfloxacin	68 (4.44)	10 (5.75)	256 (20.17)	41 (25.47)	2 (66.67)	3 (50.00)	11 (2.51)	17 (2.89)	0
Ofloxacin	290 (18.94)	75 (43.10)^ *^	298 (23.48)^ *^	122 (75.78)^ *^	2 (66.67)^ *^	2 (33.33)^ *^	32 (7.31)	256 (43.46)^ *^	1 (8.33)
Oxacillin	0	0	0	0	0	0	1 (0.23)	0	0
Pazufloxacin	0	0	0	0	0	0	0	3 (0.51)	0
Pefloxacine	0	0	0	0	0	0	0	1 (0.17)	0
Phenoxymethylpenicillin	2 (0.13)	1 (0.57)	2 (0.16)	4 (2.48)	0	1 (16.67)	1 (0.23)	1 (0.17)	0
Piperacillin	84 (5.49)	0	22 (1.73)	0	0	0	16 (3.65)	5 (0.85)	1 (8.33)
Piperacillin-Tazobactam	895 (58.46)^ *^	93 (53.45)^ *^	500 (39.40)^ *^	105 (65.22)^ *^	2 (66.67)^ *^	6 (100)^ *^	156 (35.62)^ *^	417 (70.80)^ *^	2 (16.67)
Polymyxin B	217 (14.17)	0	4 (0.32)	0	-	0	4 (0.91)	14 (2.38)	0
Rifampicin	0	0	0	0	0	0	2 (0.46)	1 (0.17)	0
Teicoplanin	6 (0.39)	0	1 (0.08)	2 (1.24)	0	0	1 (0.23)	2 (0.34)	0
Tetracycline	66 (4.31)	0	93 (7.33)	3 (1.86)	0	4 (66.67)^ *^	15 (3.42)	15 (2.55)	1 (8.33)
Ticarcillin	15 (0.98)	64 (36.78)^ *^	48 (3.78)	70 (43.48)^ *^	0	0	19 (4.34)	288 (48.90)^ *^	0
Ticarcillin-Clavulanate	20 (1.31)	1 (0.57)	3 (0.24)	0	0	0	148 (33.79)^ *^	23 (3.90)	0
Tigecycline	408 (26.65)^ *^	1 (0.57)	65 (5.12)	1 (0.62)	0	3 (50.00)^ *^	-	24 (4.07)	5 (41.67)^ *^
Tobramycin	241 (15.74)	45 (25.86)^ *^	30 (2.36)	61 (37.89)^ *^	0	0	18 (4.11)	276 (46.86)^ *^	0
Trimethoprim	112 (7.32)	3 (1.72)	43 (3.39)	0	0	0	32 (7.31)	0	2 (16.67)
Trimethoprim-Sulfamethoxazole	598 (39.06)^ *^	116 (66.67)^ *^	582 (45.86)^ *^	116 (72.05)^ *^	2 (66.67)^ *^	0	50 (11.42)	454 (77.08)^ *^	3 (25.00)^ *^
Range (%)	0-71	1-74	0-72	1-87	33-67	17-100	0-54	0-89	8-83
Average resistance (%)	21	26	16	28	55	58	15	24	30

AMR pattern of ESKAPE across the study sites

**Figure 2:**
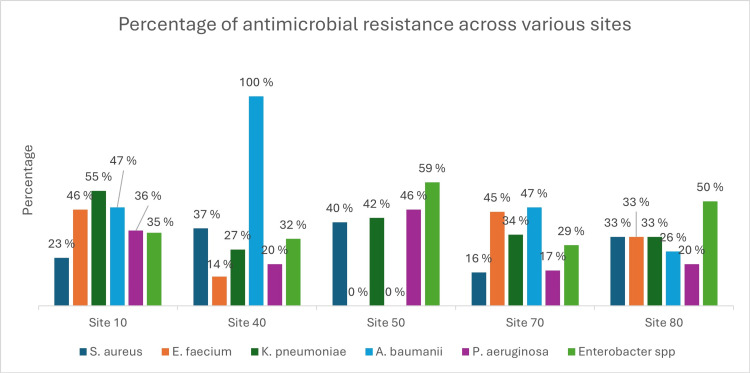
Percentage antimicrobial resistance of ESKAPE (Enterococcus faecium, Staphylococcus aureus, Klebsiella pneumoniae, Acinetobacter baumannii, Pseudomonas aeruginosa, and Enterobacter species ) across various sites

The resistance pattern of ESKAPE across the various sites showed, that S. aureus had varying levels of resistance across the sites (15.86% to 40.42%). Enterococcus faecium showed the highest at sites 10 and 70 (45.83%). Resistance pattern for K. pneumoniae ranged from 26.71% to 54.65%. Least resistance was observed in site 40 (26.71%). Site 40 shows complete resistance to A. baumannii, while other sites show varying levels of resistance, with site 80 having the lowest at 26.03%. Resistance for P. aeruginosa ranges from 17.27% to 46.25%. Enterobacter species highest resistance was observed at site 10 (59.09%) and the lowest at site 40 (31.87%) (Figure 2).

## Discussion

AMR is killing more people than cancer and traffic accidents combined, with 700,000 individuals losing the fight against it each year and another 10 million expected to do so by 2050 [14]. The World Health Organization (WHO) and several other stakeholders have become concerned about the global growth of AMR [15]. The major problem is the dearth of data and insufficient research, which makes it difficult to estimate the precise increase and scope of AMR in India and to make cross-country comparisons. 1,040 (48.3%) of 2,152 studies on AMR published by Indian institutes focused on people, compared to just 70 (3.3%) on animals and 90 (4.2%) on the environment [16].

In our study, large data (n=21,556) of various age groups were evaluated (newborn to elderly), with the mean age being 55.2 years. Due to a reduction in both quantitative and qualitative immune function as patients age, immunosenescence increases the risk of morbidity and death owing to infectious disease processes. To maintain the health of this vulnerable group, effective and safe antimicrobial treatment is crucial. We must comprehend the changed pharmacokinetics and pharmacodynamics of medicines in older patients due to comorbid illnesses and the typical physiological changes associated with aging to be able to deliver effective antibacterial treatment to this population [17].

In our study, the commonest isolated gram-positive bacterium was S. aureus, comprising more than two-thirds of gram-positive isolates. For coagulase-negative staphylococci, the commonest resistance was to phenoxymethylpenicillin followed by erythromycin; for S. aureus, resistance was to ciprofloxacin followed by erythromycin and trimethoprim-sulfamethoxazole. Enterococcus spp. also showed high resistance to erythromycin followed by ampicillin and ciprofloxacin. These findings strongly indicate that besides the methicillin class of drugs, macrolides and fluoroquinolones also became resistant to common gram-positive bacteria. According to recent publications, MDR gram-positive bacterial infections are becoming more common; MRSA among invasive S. aureus isolates was estimated to be 29% in 2009 but grew to roughly 47% in 2014 [11]. Moreover, MRSA infections, are linked to high death rates in India, in addition to delayed healing and long-term impairment [18]. In bloodstream infections, there is a strong tendency toward the growth of gram-positive bacteria, with Staphylococcus accounting for up to 27.4% of all blood culture specimen isolates [19]. The growing shortage of efficient antimicrobial medicines to treat patients with severe diseases is a direct result of the evolution of MDR among gram-positive bacteria leading to an increase in mortality, morbidity, ICU admissions, comorbidities, need for additional antimicrobial medicines, and lengthier hospital stay. While gram-negative bacteria are the most common source of ICU infections in India, gram-positive organisms such as MRSA and VRE, are also a substantial contributor to the disease. S. aureus and Enterococcus species caused 8.2% and 5.0%, respectively, of the ICU infections [20]. Another study (INDICAPS Study) conducted by Divatia et al., between 2010 and 2011 reported that gram-positive infections accounted for 15.9% of ICU infections, and gram-negative organisms at 68.9% [21].

In our study, common gram-negative species were K. pneumoniae, followed by E. coli, Acinetobacter, and P. aeruginosa. For K. pneumonia, the commonest resistance was to ciprofloxacin, cefuroxime, and cefepime. For E. coli, resistance was similar, with ampicillin followed by ciprofloxacin and cephalosporins (ceftriaxone, cefepime). For Acinetobacter spp., resistance was to ceftazidime followed by ciprofloxacin, and gentamicin while for P. aeruginosa positive samples, resistance was to imipenem and meropenem. Resistance was observed even for antibacterial agents considered last-resort drugs like carbapenems. Gram-negative bacteria acquire resistance more quickly than Gram-positive bacteria. Still, there are also fewer newly developed antibiotics that are effective against them, and drug research programs are insufficient to offer therapeutic coverage in 10-20 years. Mobile genes on plasmids that may easily circulate across bacterial populations are mostly to blame for the growth in resistance of gram-negative bacteria [22]. In India, 70%-90% of Enterobacteriaceae members have been shown to generate extended-spectrum beta-lactamases (ESBLs) in recent studies. Although this may be a skewed sample, it indicates a severe issue, necessitating widespread use of restricted antibiotics like carbapenems [23,24]. While cephalosporin resistance rates are lower in other nations, the increasing number of ESBL manufacturers is enough to increase the need for carbapenems. Thus, there is a selection pressure for carbapenem resistance in members of the family Enterobacteriaceae, and its growth is a worldwide public health problem as there are few medicines in reserve beyond carbapenems [25]. The research analyzed 2,731 isolates of gram-negative bacteria throughout the study period; 44% of E. coli, 25% of K. pneumoniae, and 11% of P. aeruginosa were identified most often. The most effective antimicrobials in the tests were carbapenems, followed by amikacin and piperacillin/tazobactam. ESBL-positive isolate rates were between 66%-77% for E. coli and 61%-72% for Klebsiella pneumonia, respectively. Overall, colistin appears promising and maintains its action in > 90% of the studied isolates. Results of this study indicate that carbapenems, amikacin, and colistin are still the best treatments for infections that are resistant. The susceptibility profile of clinically significant Gram-negative bacteria must be continuously monitored to direct successful antibiotic treatment [26].

Six nosocomial infections that show MDR and pathogenicity are ESKAPE. In our study also, isolates positive for ESKAPE organisms were high. The requirement is for novel therapeutics to treat drug-resistant infections, particularly ESKAPE pathogens. The mechanisms of MDR exhibited by ESKAPE are broadly divided into drug inactivation caused by an irreversible cleavage catalyzed by an enzyme, modification of the target site where the antibiotic may bind, and decreased accumulation of drug by reduced permeability or by increased efflux of the drug [27]. Additionally, they are known to create biofilms that guard specialized dormant cells known as persister cells that are resistant to antibiotics and create refractory infections [28]. In a study, data on antibiotic resistance in priority hospital-acquired infections (HAIs-ESKAPE) was compared between low- and lower-middle-income countries (L-LMICs) and high-income countries (HICs). Pooled methicillin resistance proportion in Staphylococcus aureus was 48.4% (95% confidence interval [95%CI] 41.7-55.2, n = 80). Pooled carbapenem resistance proportions were high in Gram-negative pathogens: E. coli: 16.6% (95%CI: 10.7-23.4, n = 60); K. pneumoniae: 34.9% (95%CI: 24.6-45.9, n = 50); P. aeruginosa: 37.1% (95%CI: 24.6-45.9, n = 56); Enterobacter spp.: 51.2% (95%CI 27.5-74.7, n = 7); and A. baumannii (complex): 72.4% (95%CI: 62.1-81.7, n = 36). Third-generation cephalosporins demonstrated higher resistance proportions. L-LMICs showed higher pooled resistance proportions for Gram-negative pathogens than regional and national estimates from HICs. Patients in resource-constrained regions are particularly affected by AMR, thus making it crucial to strengthen local surveillance and the health systems in general [29].

MDR microorganism infections are becoming an increasing concern, particularly in the intensive care unit (ICU) where even susceptible pathogens can increase morbidity, mortality, and hospital expenses. To win this battle, therefore, more work will be required in the future. To address this problematic issue, progress in current practice evaluation based on time-based MDR trends and antibiotic consumption patterns is critical.

The Government of India formulated a Core Working Group on AMR that drafted a national action plan (NAP) for the country consisting of six strategic goals that all, directly or indirectly, consider AMR in the environment [30]. Each strategic goal contains specific actions, tasks, and results, whose accomplishment is outlined by a timeframe for the next five years. The NAP is yet to be fully implemented in Indian States. The Indian drug regulator-imposed Schedule H1 in 2014, to stop the practice of over-the-counter medicine sales. However, it only includes a few antibacterial classes [11].

The patient outcomes were not evaluated prospectively for enrolled cases which can be considered a limitation. Also, we observed that a substantial number of samples did not have the date, which prevented time-based trend analysis. Nevertheless, a large number of samples evaluated across various Indian ICUs helped in understanding the evolving AMR trend which can be an important reference document for Indian clinicians and government bodies.

## Conclusions

AMR is rampant in Indian ICU patients across all age groups and genders. Staphylococci species are the most isolated gram-positive resistant bugs, resistant against multiple antibiotics like fluoroquinolones, macrolides, and penicillin. Gram-negative was more common than gram-positive species in causing antibiotic-resistant infections in ICU, with beta-lactams, fluoroquinolones, macrolides, and cephalosporins all showing varied percentages of resistance. The high prevalence of AMR necessitates strong initiatives by healthcare professionals and organizations, which can help take robust steps against AMR. This pattern of observed MDR and antibiotic consumption calls for enhanced antimicrobial surveillance and strict implementation of antimicrobial stewardship programs.
